# Supplementary figures and images for: Intragranuloma Accumulation and Inflammatory Differentiation of Neutrophils Underlie Mycobacterial ESX-1-Dependent Immunopathology
Source: mBio. 2023 Apr 5;14(2):e02764-22. doi: 10.1128/mbio.02764-22 (PMC10127687; doi:10.1128/mbio.02764-22)

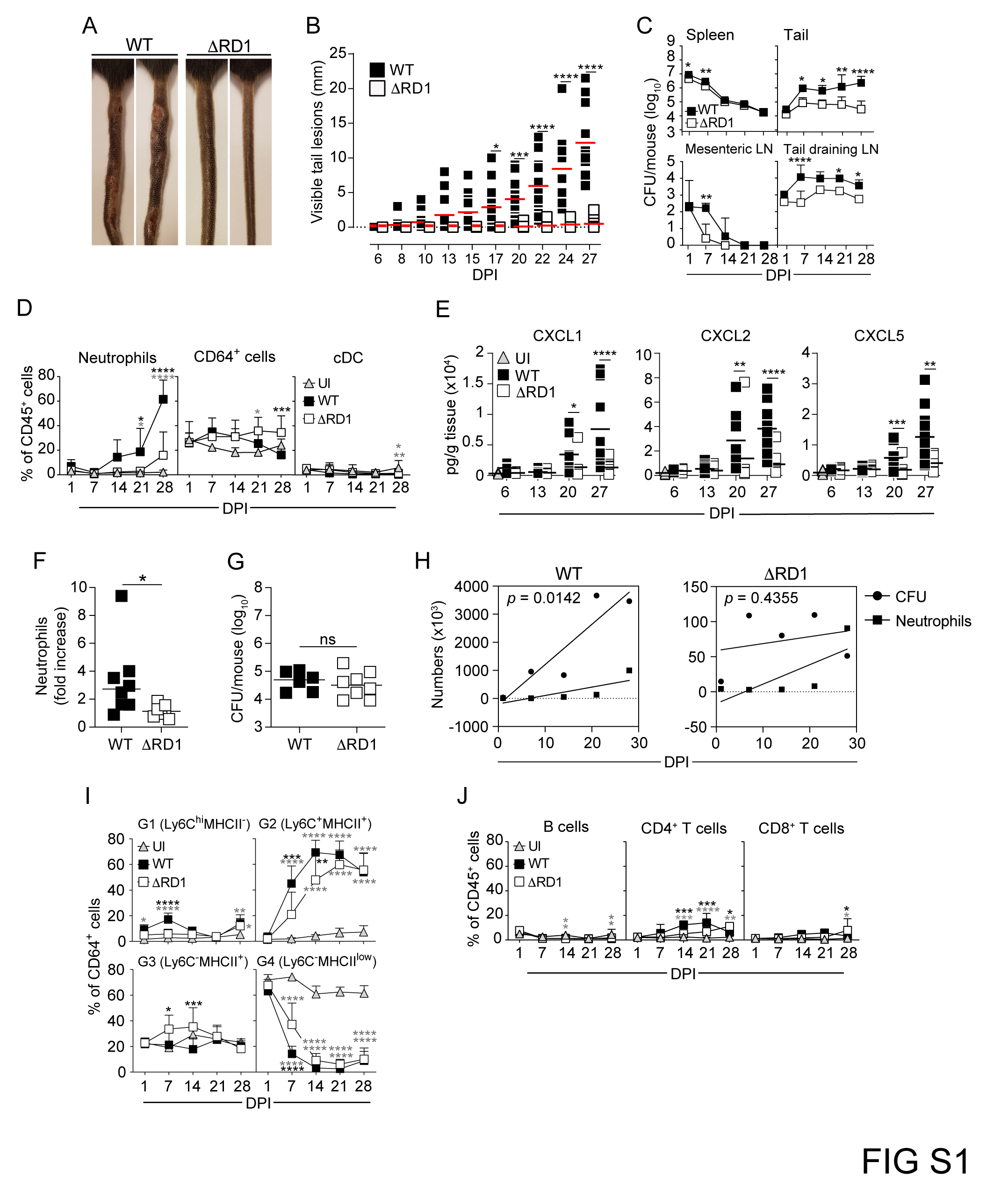

Supplement: FIG S1 [file mbio.02764-22-s0001.tif]

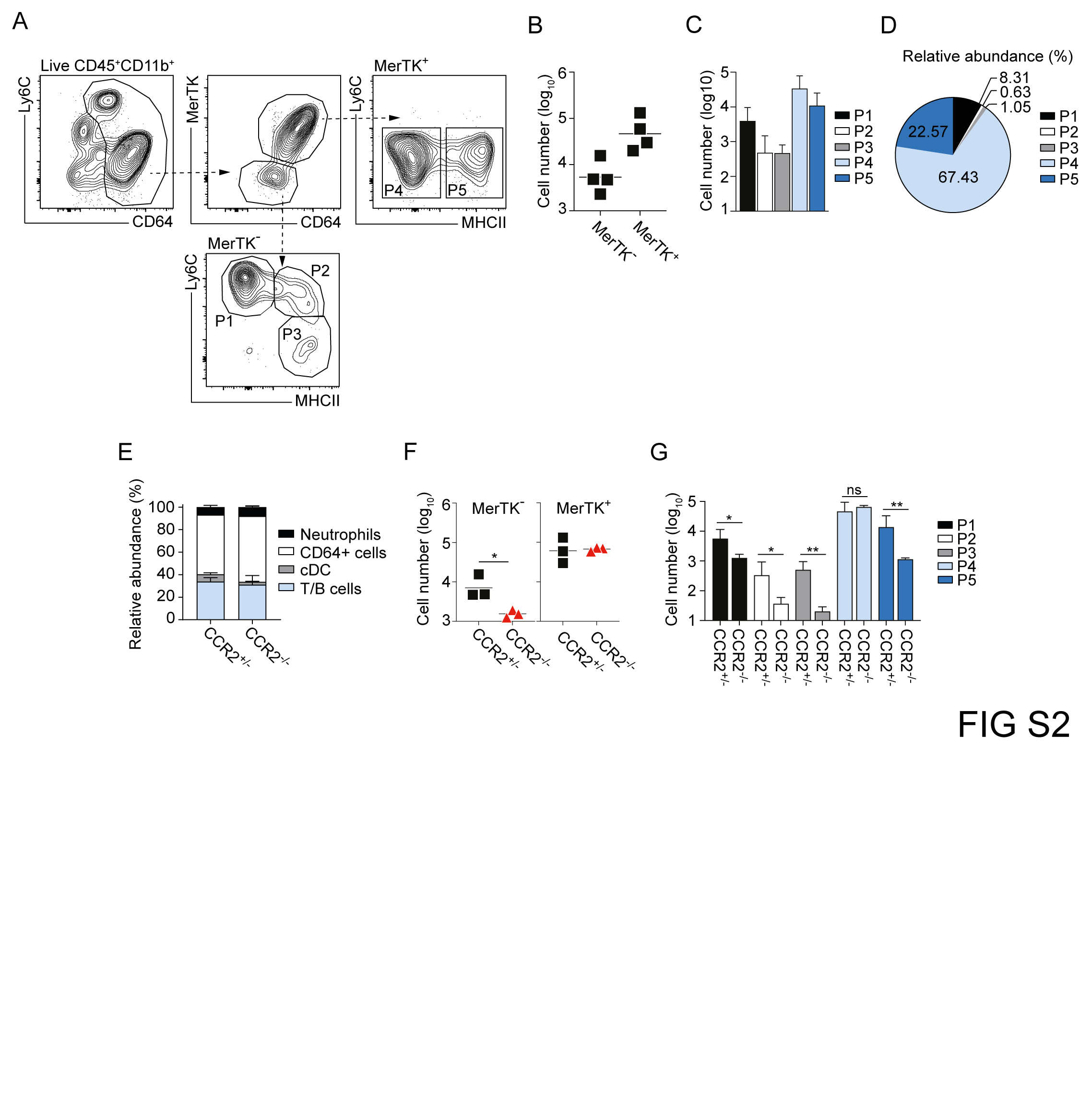

Supplement: FIG S2 [file mbio.02764-22-s0002.tif]

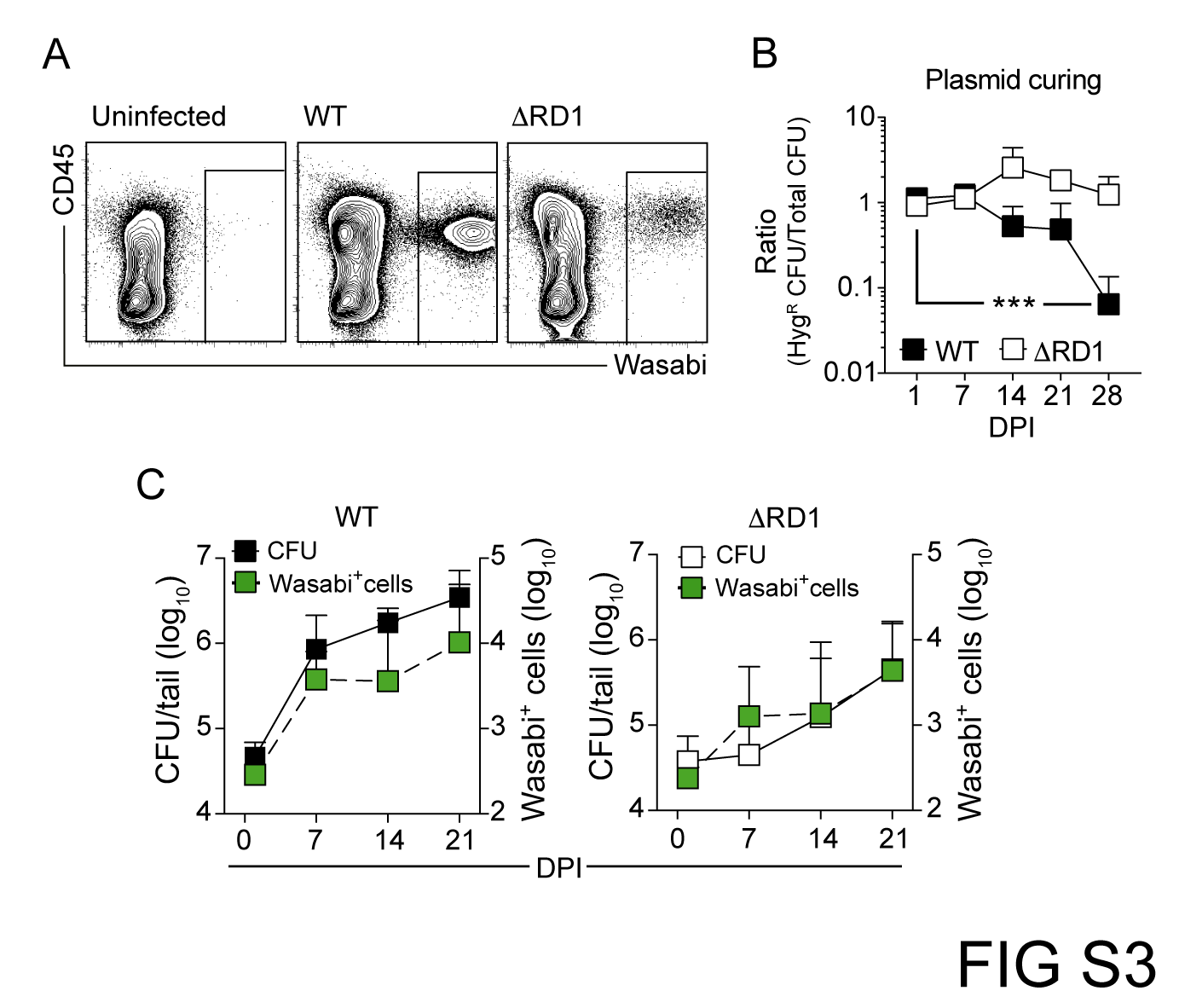

Supplement: FIG S3 [file mbio.02764-22-s0003.tif]

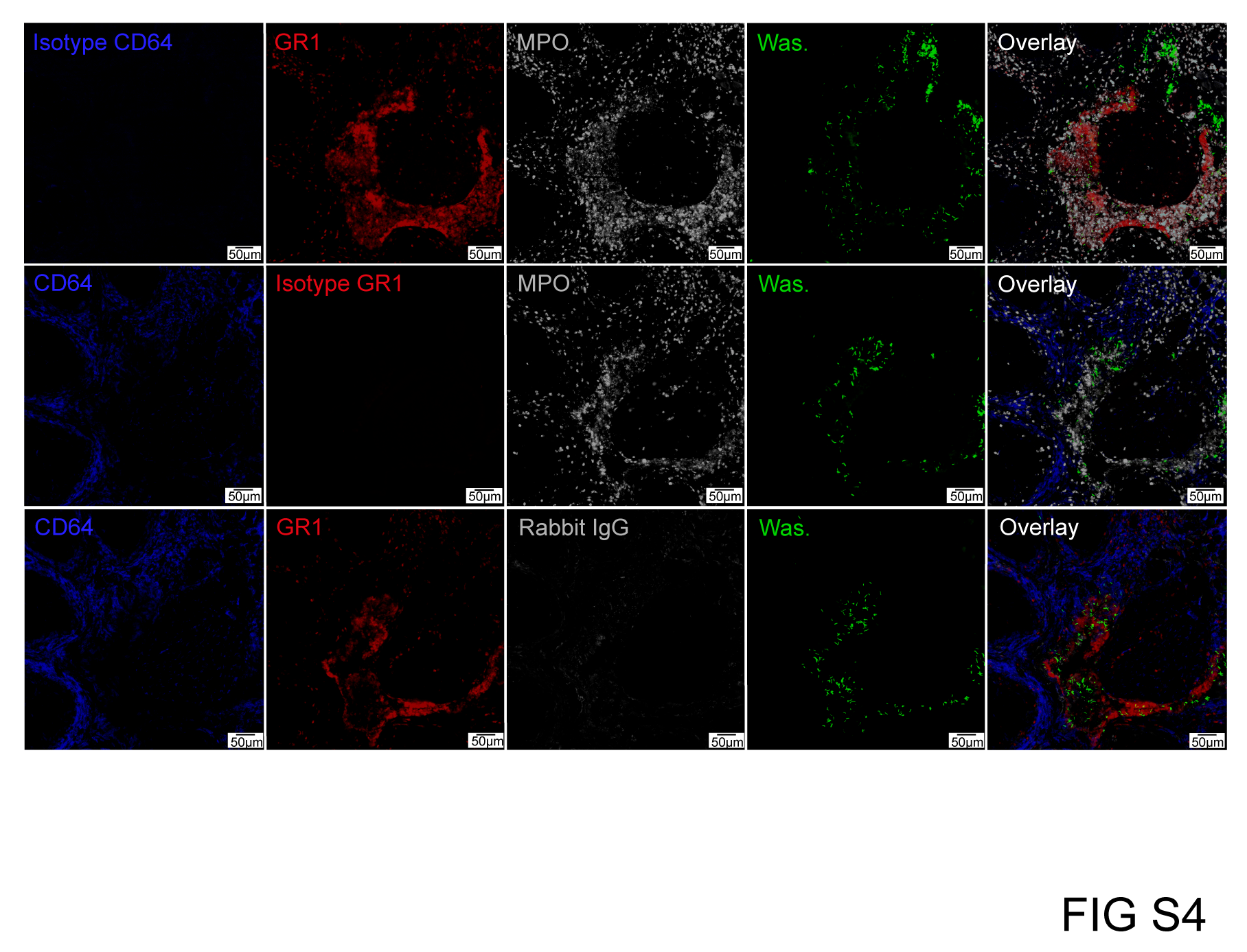

Supplement: FIG S4 [file mbio.02764-22-s0004.tif]

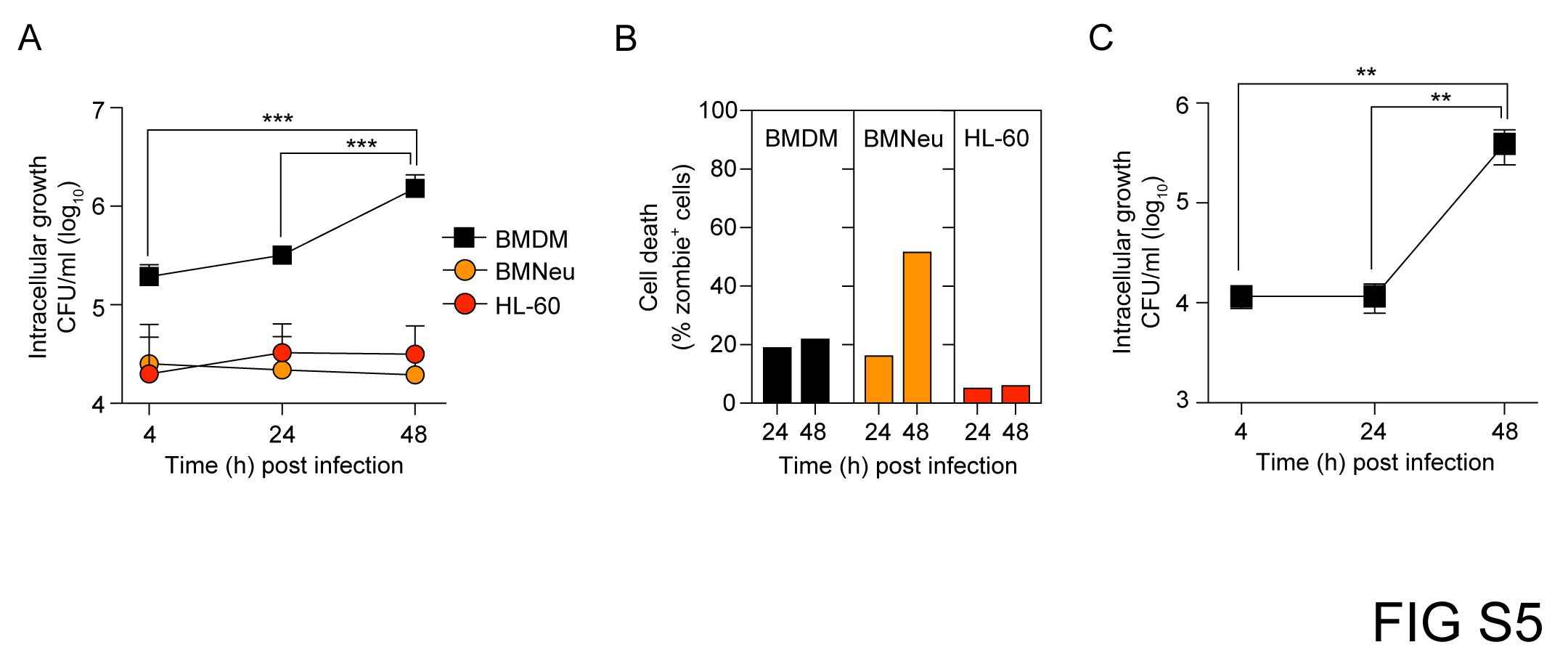

Supplement: FIG S5 [file mbio.02764-22-s0005.tif]

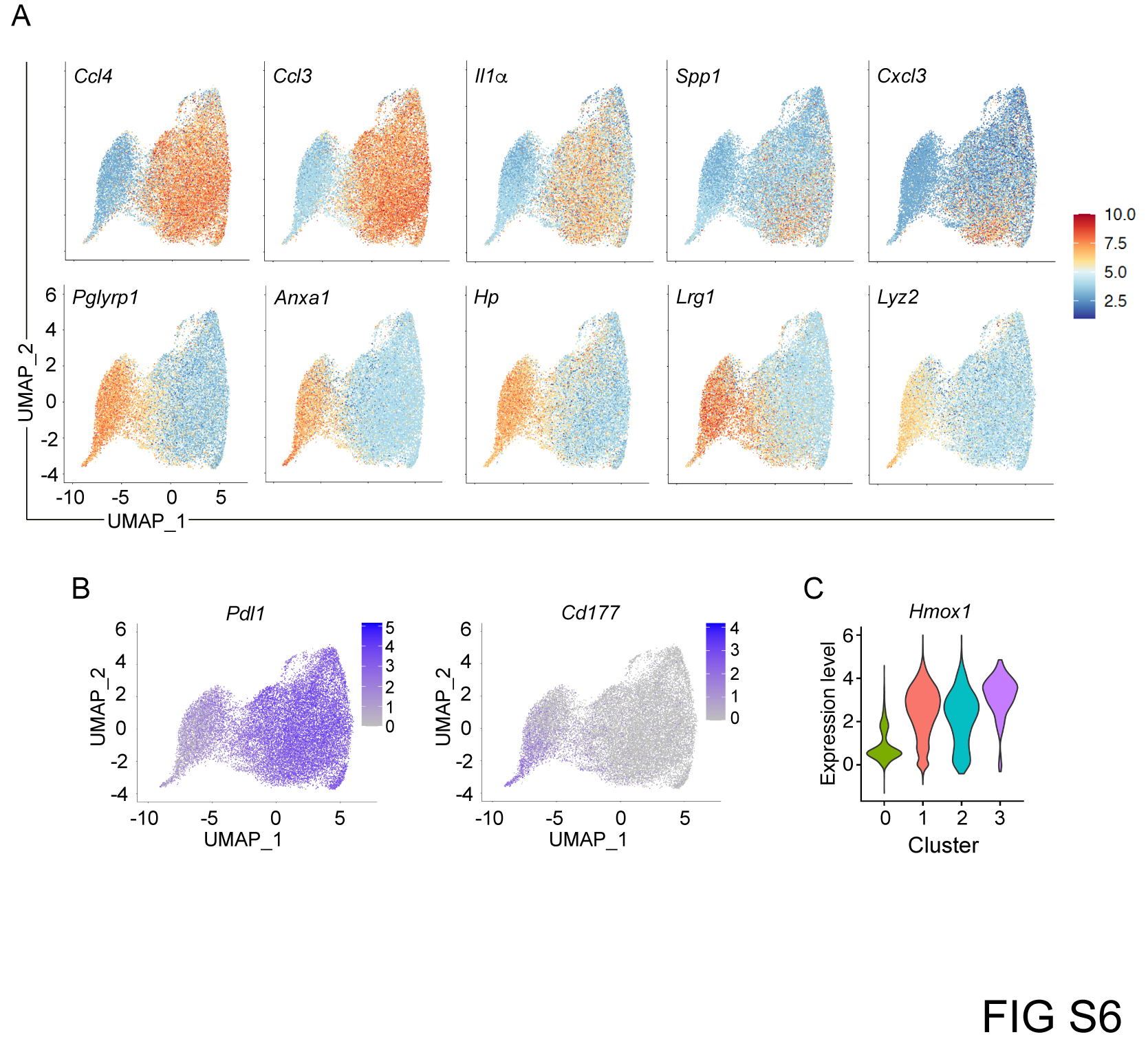

Supplement: FIG S6 [file mbio.02764-22-s0006.tif]

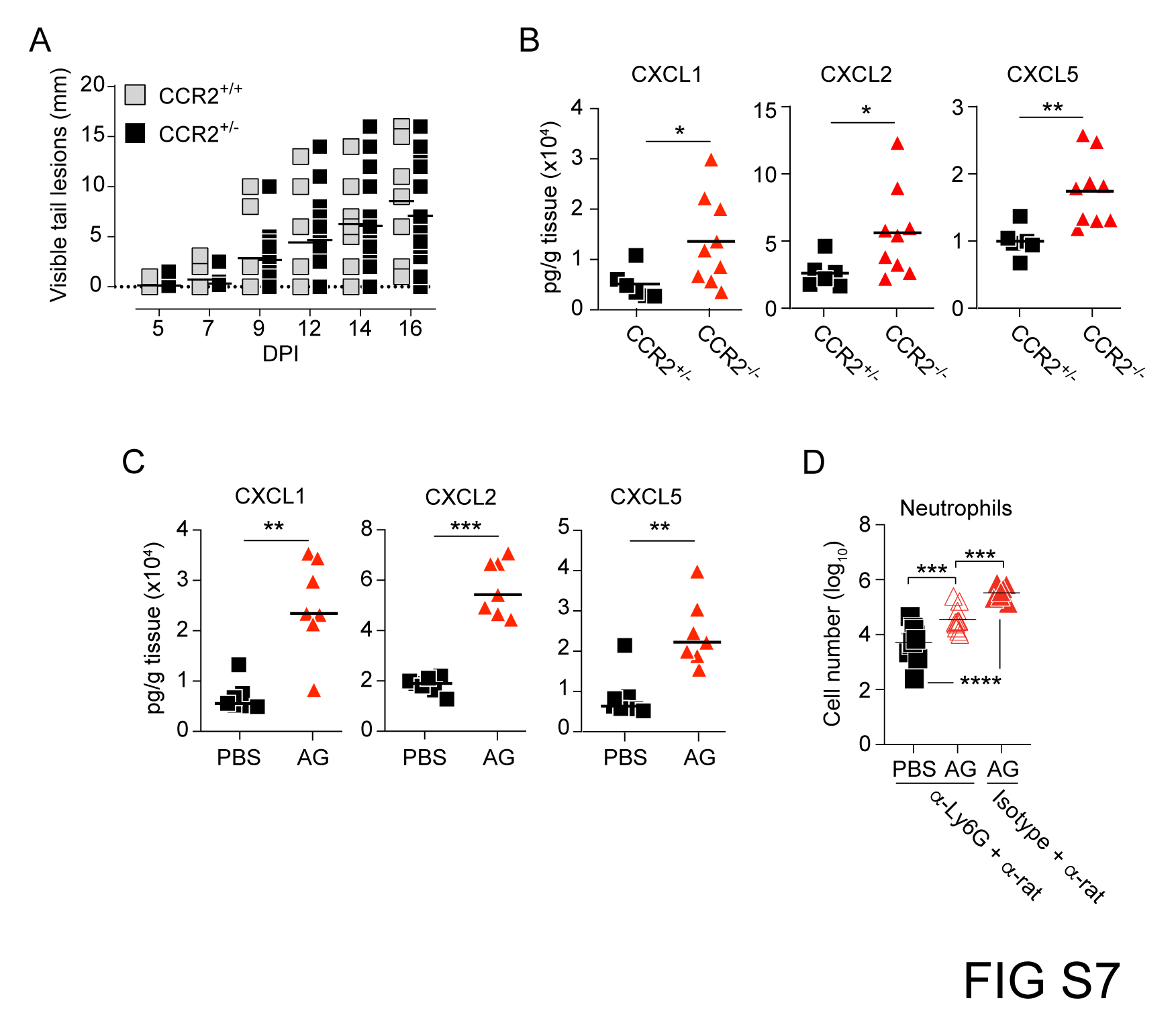

Supplement: FIG S7 [file mbio.02764-22-s0007.tif]

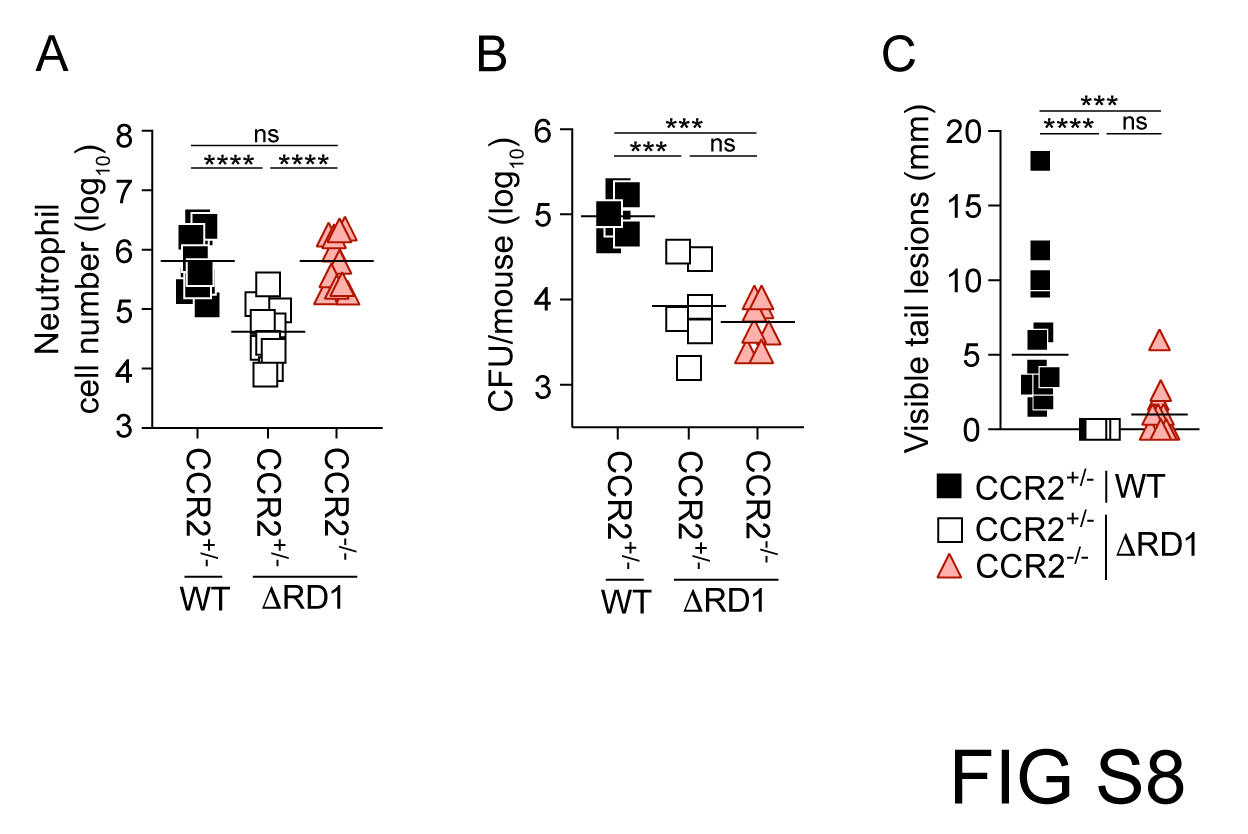

Supplement: FIG S8 [file mbio.02764-22-s0008.tif]
