# Supplementary material for: Intragranuloma Accumulation and Inflammatory Differentiation of Neutrophils Underlie Mycobacterial ESX-1-Dependent Immunopathology
Source: mBio. 2023 Apr 5;14(2):e02764-22. doi: 10.1128/mbio.02764-22 (PMC10127687; doi:10.1128/mbio.02764-22)
Supplement: TABLE S2 [file mbio.02764-22-s0010.pdf]

Table S2. Detailed list of the materials and reagents used.

Antibodies	Source	Reference
Monoclonal rat anti-CD3	BioLegend	CAT#100227 and CAT#100216, clone 17A2
Monoclonal rat anti-CD4	BioLegend	CAT#100545, clone RM4-5
Monoclonal rat anti-CD8a	BD Biosciences	CAT#563332, clone 53-6.7
Monoclonal rat anti-CD11b	BioLegend	CAT#101237, clone M1/70
Monoclonal rat anti-CD11b	eBiosciences	CAT#47-0112-82, clone M1/70
Monoclonal Armenian hamster anti-CD11c	eBiosciences	CAT#25-0114-82, clone N418
Monoclonal rat anti-CD19	BioLegend	CAT#115554 and CAT#115537, clone 6D5
Monoclonal rat anti-CD19	eBiosciences	CAT#56-0193-82, clone 1D3
Monoclonal mouse anti-CD45.2	eBiosciences	CAT#56-0454-82, clone 104
Monoclonal rat anti-CD45.2	BioLegend	CAT#103137, clone 30-F11
Monoclonal mouse anti-CD64	BD Biosciences	CAT#558539, clone X54-5/7.1
Monoclonal mouse anti-CD64	BioLegend	CAT#139308, clone X54-5/7.1
Monoclonal rat anti-CD115	BioLegend	CAT#135528, clone AFS98
Monoclonal rat anti-Gr-1	BioLegend	CAT#108416 and CAT# 108430, clone RB6-8C5
Monoclonal rat anti-Ly6C	BioLegend	CAT#128012 and CAT#128041, clone HK1.4
Monoclonal rat anti-Ly6G	BD Biosciences	CAT#563979, clone 1A8
Monoclonal rat anti-Ly6G	BioLegend	CAT#127628 and CAT#127645, clone 1A8
Monoclonal rat anti-MHCII/I-A/I-E	BD Biosciences	CAT#563415, clone M5/114.15.2
Monoclonal rat anti-MHCII/I-A/I-E	BioLegend	CAT#107635, clone M5/114.15.2
Monoclonal Armenian Hamster anti-TCRb	BioLegend	CAT#109230, clone H57-597
Monoclonal Armenian Hamster anti-TCRb	BD Bioscience	CAT#562841, clone H57-597
Monoclonal mouse anti-Nos2	Santa Cruz Biotechnology	CAT#sc-7271, clone C-11
Polyclonal rabbit anti-MPO	Thermo Fischer	CAT#PA5-16672
Monoclonal mouse IgG1k isotype control	BD Biosciences	CAT#565571, clone MOPC-21
Monoclonal rat anti-FC (CD16/CD32) block	BD Biosciences	CAT#553142, clone 2.4G2
Polyclonal alpaca anti-rabbit	Jackson ImmunoResearch	CAT#611-585-215
Monoclonal rat *InVivo*MAb anti-Ly6G	BioXell	CAT#BE0075-1, clone 1A8
Monoclonal rat *InVivo*MAb anti-trinitrophenol isotype control	BioXell	CAT#BE0089, clone 2A3
Monoclonal mouse *InVivo*MAb anti-rat	BioXell	CAT#BE0122, clone MAR 18.5
Monoclonal mouse IgG1k isotype control	BD Biosciences	CAT#557732, clone MOPC-21
Monoclonal rat IgG2bk isotype control	BioLegend	CAT#400639, clone RTK4530

Preimmune polyclonal rabbit IgG	Sigma-Aldrich	CAT#I5006
***Mycobacterium marinum* strains**	**Reference**	
Wild type (WT)	Volkman *et al.* PLoS Biol 2004	
ΔRD1	Volkman *et al.* PLoS Biol 2004	
**Cell lines**	**Reference**	
Human HL-60 cell line	ATCC: CCL-240	
**Commercial Assays**	**Source**	**Reference**
LIVE/DEAD™ Fixable Near-IR Dead Cell Stain Kit	Thermo Fischer	CAT#L34975
Mouse CXCL1/KC ELISA kit	R&D systems	CAT#DY453-05
Mouse CXCL2/MIP-2 ELISA kit	R&D systems	CAT#DY452-05
Mouse CXCL5/LIX ELISA kit	R&D systems	CAT#DY443
GoScript Reverse Transcription System	Promega	CAT#A5001
SsoFast EvaGreen qPCR super mix	BioRad	CAT#172-5204
RNeasy Mini Kit	Qiagen	CAT#74106
Chromium Single Cell 3′ Library & Gel Bead Kit v3	10x Genomics	CAT#PN-1000092
Chromium Chip B Single Cell Kit	10x Genomics	CAT#PN-1000074
Chromium Controller & Next GEM Accessory Kit	10x Genomics	CAT#PN-120223
Dynabeads MyOne silane	10x Genomics	CAT#PN-2000048
SPRIselect Reagent kit	Beckman Coulter	CAT#B23318
Chromium i7 Sample Index	10x Genomics	CAT#PN-220103
KAPA Library Quantification Kit for Illumina Platforms	Kapa Biosystems	CAT#KK4873
2100 Bioanalyzer equipped with a High Sensitivity DNA kit	Agilent	CAT#5067-4626
Zombie Aqua Fixable Viability kit	Biolegend	CAT#423101
**Reagents/chemicals**	**Source**	**Reference**
DPBS	Gibco™/LifeTechnologies	CAT#14190-144
RPMI Medium 1640	Gibco™/LifeTechnologies	CAT#31870-025
Hygromycin B	Invitrogen	CAT#10687010
Middlebrook 7H9	BD Biosciences	CAT#271310
Middlebrook 7H10	BD Biosciences	CAT#262710
ACD enrichment for 7H9	BD Biosciences	CAT#212352
OADC enrichment for 7H10	Conda Lab	CAT#6037
cOmplete™, EDTA-free Protease Inhibitor Cocktail)	Roche	CAT#11873580001
Ultra pure 0.5M EDTA, pH8	Invitrogen	CAT#15575-038
Amikacin	Sigma-Aldrich	CAT#A3650
ProLong™ Gold Antifade Mountant	Invitrogen	CAT#P36930
Glycerol	Sigma-Aldrich	CAT#G6279
Tween 80	Sigma-Aldrich	CAT#P1754
Triton X100	Thermo Fisher	CAT#T8787
AntigenFix	Diapath	CAT#P0014
Aminoguanidine (AG) hemisulfate salt	Sigma-Aldrich	CAT#A7009
Liberase TM	Roche	CAT#05401127001
DNAse I	Sigma-Aldrich	CAT#10104159001
N,N-dimethylformamide (DMF)	Fisher Scientific	CAT#D131-1
Paraformaldehyde (PFA)	Alfa Aesar/Thermo Fisher	CAT#J61899
Fetalclone I	Hyclone	CAT#SH30080.03
Histopaque 1077/1119	Sigma-Aldrich	CAT#10771/11191-100mL

L-Glutamine 200mM (100X)	Gibco™/LifeTechnologies	CAT#25030-024
AccCount Fluorescent Particles	Spherotech	CAT#ACFP-70-10
Saponin	Sigma-Aldrich	CAT#8047-15-2
Sucrose	Sigma-Aldrich	CAT#84100-1KG
FBS	Sigma-Aldrich	CAT#F7524
BSA	Sigma-Aldrich	CAT#A7906
Propidium Iodide (PI)	Invitrogen	CAT#P3566
**Plasmids**	**Source**	**Reference**
pTEC15 plasmid (Wasabi)	Addgene	CAT#30174
**Primers**	**Sequence**	
Nos2 forward	5´-TGGAGCGAGTTGTGGATTGTC	
Nos2 reverse	5´-GGGCAGCCTCTTGTCTTTGA	
reep5 forward	5´-GATACCCAGCCTACATCTCAATG	
reep5 reverse	5´-GCAATGCTGAACACACCATATAC	
**Equipment**	**Source**	
Flow cytometer LSRII	BD Biosciences	
FACS ARIA Fusion	BD Biosciences	
Biopulverizer	Biospec Products	
Homogenizer PT 1200 E	Polytron	
CFX 384 Real-Time PCR Detection System	Biorad	
Nanodrop ND1000	Thermo Fisher	
TissueLyser II	Qiagen	
CM1950 cryostat	Leica	
VS-120 virtual slide scanning microscope	Olympus	
**Software and Algoritms**	**Version**	
FlowJo (BD)	version 9.9.6 and v10.7.1	
FACSDiva (BD)	version 8.0	
CFX Maestro (Biorad)	version 2.0	
Photoshop (Adobe)	2020/2021	
VS-ASW-S6 software (Olympus)	version 2.9	
Prism (GraphPad)	version 8	
CellRanger (10X Genomics)	version 2.1.1 and v3.1.0	
R	version 3.5.1 and v4.0.2	
velocyto R	version 0.6 (using R version 3.5.1)	
Seurat	version 3.1.5	
Anaconda3	version 4.4.0	
